# Human papillomavirus persistence and clearance in women living with HIV: A five‐year retrospective analysis from an Italian university center

**DOI:** 10.1111/aogs.70299

**Published:** 2026-06-25

**Authors:** Annunziata Carlea, Dario Colacurci, Giorgia D'Apice, Laura Letizia Mazzarelli, Massimiliano Pellicano, Luigi Falco, Ginevra Sepe, Oriana Imperatore, Giuseppe Maria Maruotti, Maurizio Guida, Laura Sarno

**Affiliations:** ^1^ Department of Neurosciences, Reproductive Science and Dentistry University of Naples Federico II Naples Italy; ^2^ Department of Public Health University of Naples Federico II Naples Italy; ^3^ Mother and Child Department University Hospital Federico II Naples Italy

**Keywords:** clearance, human papillomavirus, women living with HIV

## Abstract

**Introduction:**

Human papillomavirus infection is one of the most prevalent sexually transmitted infections worldwide. Women living with HIV are at increased risk of acquiring and developing persistent infection with high‐risk HPV genotypes, leading to higher rates of cervical dysplasia and cancer. However, limited data are available regarding the timing and determinants of HPV clearance in this population.

**Material and Methods:**

This is a retrospective, single‐center study including women living with HIV with confirmed high‐risk HPV infection, followed at an Italian university hospital between 2019 and 2024. Clinical, virological, and immunological data were collected, including HIV viral load, CD4+ T‐cell count, and adherence to antiretroviral therapy. HPV persistence, clearance, and time to clearance were assessed over a 5‐year follow‐up period. Statistical analyses were performed using SPSS version 29, with *p* < 0.05 considered significant.

**Results:**

Seventy‐seven women living with HIV were recruited, and fifty‐one met the inclusion criteria for analysis. Most participants (92%) were adherent to ART. HR‐HPV clearance occurred in 76.5% of patients, with a mean clearance time of 2.08 years. No significant correlation was observed between HPV clearance time and HIV viremia, CD4+ T‐cell count, or cytological/colposcopic findings at baseline. However, baseline positivity for the high‐risk HPV pool was significantly associated with longer clearance time (*p* = 0.011).

**Conclusions:**

Women living with HIV showed a high‐risk HPV clearance time of approximately 2 years under ART. Our findings suggest that HPV screening every 2 years may represent an appropriate interval in this population, potentially increasing adherence and optimizing healthcare resources. Larger multicenter prospective studies are needed to confirm this observation.

## Graphical abstract

In women living with HIV, HPV clearance occurred after a mean of approximately 2 years under antiretroviral therapy. Baseline positivity for the high‐risk HPV pool was associated with delayed clearance, suggesting that HPV genotype may influence viral persistence more than immunovirological status in clinically stable patients.

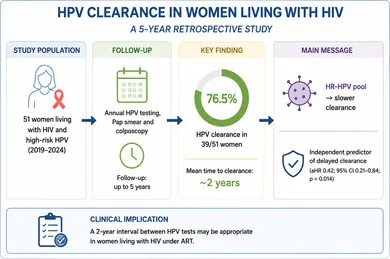

**Abbreviations**
ART: Antiretroviral Therapy
ASC‐US: Atypical Squamous Cells of Undetermined Significance
CIN: Cervical Intraepithelial Neoplasia
DNA: Deoxyribonucleic Acid
HAART: Highly Active Antiretroviral Therapy
HIV: Human Immunodeficiency Virus
HPV: Human Papillomavirus
HR‐HPV: High‐Risk Human Papillomavirus
PCR: Polymerase Chain Reaction
RNA: Ribonucleic Acid
WLWH: Women Living with HIV

**Key message**

In women living with HIV with good adherence to antiretroviral therapy, high‐risk HPV infection clears in approximately 2 years. Baseline HPV genotype—rather than HIV‐related immunovirological status—appears to be the main determinant of clearance timing, supporting a 2‐year HPV screening interval in this population.

## 1 INTRODUCTION

Human papillomavirus (HPV) infection is worldwide considered to be one of the most common sexually transmitted diseases, and it is considered a significant public health problem.^1^ HPV infection is highly prevalent among sexually active individuals worldwide.^2^ More than 200 HPV types have been identified, of which approximately 40 infect the anogenital tract and oropharyngeal mucosa.^3^, ^4^, ^5^ HPV genotypes are broadly classified into high‐risk (HR‐HPV) and low‐risk types according to their oncogenic potential.^6^, ^7^, ^8^ Low‐risk types are typically associated with benign lesions, whereas HR‐HPV genotypes are responsible for high‐grade intraepithelial lesions and invasive cancers.^9^ Among these HR‐HPV genotypes, genotypes 16 and 18 are highly oncogenic and account for most cervical cancer‐associated HPVs,^10^ while other genotypes contribute to a smaller proportion.^11^ HR‐HPV infection is considered a necessary cause of almost all cervical cancers.^12^ This relationship is particularly relevant in women living with HIV (WLWH), in whom the risk of HPV persistence and progression to cervical dysplasia and cancer is significantly increased.^12^ It is also associated with a subset of cancers of the vulva, vagina, and penis.^13^ There is a strong bidirectional link between human papillomavirus (HPV) and human immunodeficiency virus (HIV) infections.^14^ HPV infection may facilitate HIV acquisition through mucosal inflammation and epithelial disruption, while HIV‐related immunosuppression significantly alters the natural history of HPV infection.^15^ Women living with HIV (WLWH) represent a particularly vulnerable population with respect to HPV infection and its clinical consequences. HIV‐related immune suppression profoundly alters the natural history of HPV infection, leading to higher rates of acquisition, reduced viral clearance, increased persistence, and a greater risk of progression to cervical intraepithelial neoplasia (CIN) and invasive cervical cancer.^16^, ^17^ Moreover, WLWH are more frequently infected with multiple HPV genotypes and exhibit a higher prevalence of HR‐HPV compared with HIV‐negative women. In contrast, HPV clearance is severely impaired among WLWH,^18^ resulting in an increased burden of HPV‐related diseases, particularly cervical dysplasia and cancer. Despite the implementation of cervical cancer screening programs, WLWH continue to experience a disproportionately high burden of HPV‐related disease. In this population, HPV persistence rather than acquisition appears to play a key role in disease progression. It is not known whether different HR‐HPV types may have different characteristics concerning persistence, progression, or oncogenicity in the context of HIV infections. A better understanding of HPV persistence and clearance dynamics in WLWH is essential to optimize screening strategies, risk stratification, and clinical management in this high‐risk group. Indeed, the primary aim of this study is to analyze the natural history of HPV infection in WLWH, with particular focus on the timing of HPV clearance and potential risk factors associated with this process.

## 2 MATERIAL AND METHODS

### 2.1 Study design and setting

This retrospective, single‐center observational study was conducted at the Department of Obstetrics & Gynecology of the University of Naples “Federico II,” Italy. The study included women living with HIV (WLWH) with high‐risk HPV (HR‐HPV) positivity detected between January 2019 and December 2024. The aim was to investigate HPV persistence and clearance, as well as potential determinants of viral persistence in this population.

### 2.2 Study population

The study population consisted of WLWH with HR‐HPV positivity who attended routine gynecological follow‐up at our institution during the study period. Inclusion criteria were: confirmed HIV infection (with or without highly active antiretroviral therapy, HAART); positive HR‐HPV DNA test; at least one follow‐up HPV test after 12 months; availability of colposcopic examination (with or without biopsy); and written informed consent for data use. Exclusion criteria were: absence of HIV infection; absence of HR‐HPV positivity; loss to follow‐up; diagnosis of invasive cervical cancer; lack of Pap smear or colposcopy; and lack of consent for data use.

### 2.3 Intervention and diagnostic procedures

**FIGURE 1:**
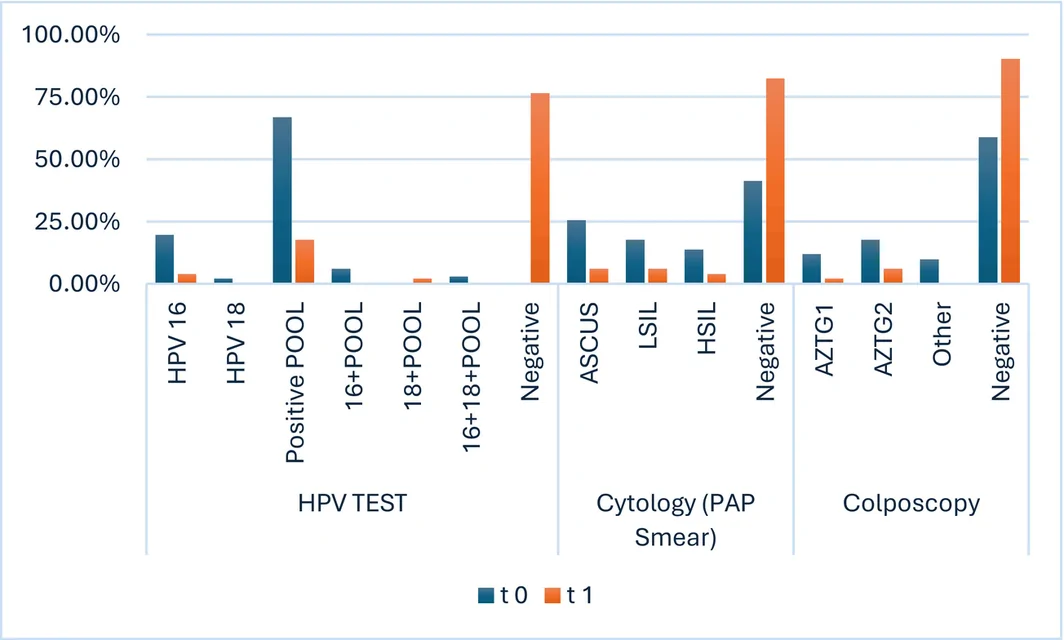
HPV TEST, cytology, and colposcopy at 5‐years follow up.

All patients underwent standard gynecological evaluation, including HPV testing, Pap smear, and colposcopy when indicated. HPV testing was performed using a polymerase chain reaction (PCR)‐based assay for HPV DNA detection. The HR‐HPV genotypes investigated included types 16, 18, 31, 33, 35, 39, 45, 51, 52, 56, 58, 59, 66, 67, and 68, in accordance with national guidelines^19^ and reflecting the range of genotypes detected by the PCR assay used in routine clinical practice. After speculum insertion, cervical samples were collected using a cervical swab. A baseline positive HPV test was defined as the first HR‐HPV‐positive result at enrollment (time zero). HPV testing was performed annually over a 5‐year follow‐up period. HPV persistence was defined as a positive HR‐HPV test at least 12 months after baseline. HPV clearance was defined as the first negative HR‐HPV test during follow‐up. A new HPV infection was defined as the detection of a new HR‐HPV genotype not present at baseline. A summary of HPV testing, cytology, and colposcopy findings during the 5‐year follow‐up is provided in Figure 1.

### 2.4 Study outcomes

The primary outcome was time to HPV clearance in WLWH. Secondary outcomes included HPV persistence, incidence of new HPV infections, and identification of clinical and immunological predictors of persistence, including CD4+ T‐cell count, HIV viral load, and HAART.

### 2.5 Data collection

Clinical and laboratory data were retrospectively collected from electronic medical records. A dedicated database was used to collect all the data. Collected variables included demographic data, HPV test results, Pap smear and colposcopy findings, previous cervical procedures, HIV viral load (RNA copies/mL), CD4+ T‐cell count, HAART regimen, and presence of other sexually transmitted infections. Data were collected independently by two researchers and verified for accuracy. The data were checked for accuracy and correctness. Each subject was assigned a numerical code to ensure anonymity and confidentiality.

### 2.6 Statistical analyses

Statistical analysis was performed using IBM SPSS Statistics software version 29. Categorical variables were defined as absolute numbers with percentages. Chi‐squared test defined associations between categorical variables. Continuous variables were inserted as means plus standard deviations (SD). Analysis of variance (ANOVA) helped to compare continuous variables with categorical ones. *p*‐value < 0.05 was considered statistically significant.

## 3 RESULTS

### 3.1 Study population

A total of seventy‐seven women living with HIV (WLWH) who tested positive for HR‐HPV were identified between 2019 and 2024, of whom fifty‐one met the inclusion criteria for analysis. The mean age of the study population was 44.6 ± 9.8 years (range 25–63). Most participants (47/51, 92.1%) were adherent to antiretroviral therapy (ART), primarily including regimens with Truvada, Lamivudine, Emtricitabine, Ritonavir, Tenofovir, or Raltegravir. HIV viral suppression (<40 copies/mL) was achieved in 90.2% of patients, while 9.8% had detectable viremia (>40 copies/mL).

Data on immunological status were available for 38 women (74.5%): 33.3% had CD4+ T‐cell counts above 500 cells/μL, whereas 41.2% had counts below this threshold. More than half of the study population (56.9%) reported active smoking at the time of enrollment.

Overall, this cohort represented a group of well‐controlled HIV‐positive women under long‐term ART, with predominantly preserved immune function.

### 3.2 Baseline HPV, cytology, and colposcopy findings

At baseline (time 0), all women had a positive HR‐HPV DNA test, including HPV 16, HPV 18, and the high‐risk HPV pool. The high‐risk HPV pool was detected in 34 patients (66.7%). All women practiced Pap smear and colposcopic examination at enrollment. Regarding cytological results, 41.2% of patients showed negative cytology. In 25.5% of cases, atypical squamous cells of undetermined significance (ASC‐US) were identified. Colposcopy was negative in 58.8% of patients. A detailed summary of HPV genotypes, cytological results, and colposcopic findings at baseline is presented in Table 1.

**TABLE 1:** Timing of HR‐HPV clearance during the 5‐year follow‐up period.

Year	*t*_1_	*t*_2_	*t*_3_	*t*_4_	No clearance at 5 years
Patients (%)	11 (21.6%)	16 (31.4%)	10 (19.6%)	2 (3.9%)	12 (23.5%)
Average	44.96		2.08		
Standard deviation	9.447		0.870		

### 3.3 Follow‐up findings and HPV clearance

During follow‐up, patients underwent a first control visit (time 1), scheduled 1 year after the baseline positive HPV test (with some variability in less compliant patients, up to 2 years). At time 1, HPV testing, Pap smear, and colposcopy were repeated according to clinical protocols. At this first follow‐up visit, HR‐HPV clearance was observed in 39 of 51 patients (76.5%). The mean time to clearance was 2.08 years, with individual variations depending on viral genotype and immune parameters.

### 3.4 Correlations and predictors of HPV clearance

The Kaplan–Meier analysis demonstrated a significant difference in the kinetics of viral clearance based on the baseline HPV genotype (log‐rank *p* = 0.011). The median time to clearance was significantly longer for women positive for the high‐risk HPV pool compared to those with HPV 16 or 18. In the multivariable Cox regression model, baseline positivity for the HR‐HPV pool remained a significant independent predictor of delayed clearance (HR 0.42; 95% CI 0.21–0.84; *p* = 0.014). Conversely, no significant associations were found between the speed of clearance and immunovirological parameters, including CD4+ T‐cell count (*p* = 0.650) and HIV viral suppression (*p* = 0.780), nor with smoking status (*p* = 0.421). Table 2 shows Cox proportional hazards regression analysis for factors associated with HR‐HPV clearance.

**TABLE 2:** Cox proportional hazards regression analysis for factors associated with HR‐HPV clearance.

Variable	Hazard ratio (HR)	95% confidence interval (CI)	*p*‐value
HPV genotype (HR‐pool vs. 16/18)	0.42	0.21–0.84	**0.014**
Age (>45 vs. ≤45 years)	1.08	0.58–2.01	0.812
CD4+ count (>500 vs. <500 cells/μL)	1.15	0.62–2.12	0.650
HIV viral load (detectable vs. undetectable)	0.88	0.35–2.21	0.780
Smoking status (yes vs. no)	0.76	0.39–1.48	0.421

*Note*: Bold value: significative results.

## 4 DISCUSSION

In this study, we included women living with HIV (WLWH) who tested positive for HR‐HPV and were monitored at the Department of Obstetrics and Gynecology of the University of Naples Federico II between 2019 and 2024. In our study population, 66.7% of patients tested positive for HR‐HPV genotypes. HPV clearance during the observation period occurred in 76.5% of the patients, with a mean clearance time of approximately 2 years. The Kaplan–Meier analysis revealed a significant difference in HPV clearance kinetics based on HR‐HPV genotypes positive at study entry (log‐rank *p* = 0.011). In particular, patients positive for the HR‐HPV pool showed a significantly slower rate of HPV clearance compared with patients positive for genotype 16 or 18. To date, few studies have analyzed the rate of HPV clearance in HIV‐positive women with good adherence to ART. Most of the available literature^20^ has demonstrated a significant increased risk of HPV persistence and progression to high‐grade lesions in individuals infected with HIV. Among immunocompetent individuals, it has been estimated that only a small percentage of HPV infections become persistent, especially those caused by HR oncogenic genotypes.^21^ The rate of HPV clearance is directly related to the cell‐mediated immune response of the host. Early initiation of ART in HIV‐infected individuals has been proven to play a crucial role in reducing the risk of HPV persistence and subsequent HPV‐related malignancies.^22^ However, our multivariable Cox regression analysis revealed that HR‐HPV positivity at study entry was the sole independent predictor of delayed HPV clearance (aHR 0.42; 95% CI 0.21–0.84; *p* = 0.014), even after adjusting for confounding variables such as age and smoking status.

Many studies have suggested that successful HIV viral suppression and higher CD4+ T‐cell counts are associated with decreased HPV replication and persistence.^23^ Interestingly, our results failed to demonstrate a significant correlation of immunovirological parameters (CD4+ T‐cell count and HIV viremia) with the time to clearance of HPV. This lack of significance should be considered with caution; considering that the majority of our population achieved viral suppression, it is important to define a possible Type II error because of the small sample size. Other individual factors that influence viral dynamics after ART initiation, including HLA haplotypes,^24^ smoking habits, and co‐infections, may also modulate viral dynamics following ART initiation. A study by Adebamowo et al.^25^ involving 630 HIV‐positive and HIV‐negative women in Nigeria showed that the clearance of HPV was not directly correlated with HIV status; however, WLWH showed a lower likelihood of clearing multiple HR‐HPV.

Coherent with our results, previous studies have reported that HPV type 16 is not the predominant type in HIV‐positive patient women.^26^ Instead, various HR‐HPV and LR‐HPV types, particularly HPV‐18 and HPV‐45, are commonly found, especially in women with HIV infection and high‐grade cervical intraepithelial neoplasia (CIN III).

The observation that the HR‐HPV pool carries a higher risk of persistence in our cohort compared to HPV 16/18 is particularly relevant. This defines that in WLWH with stable immune function, the clinical focus should remain high for the entire HR‐HPV spectrum, as non‐16/18 types may exhibit a more indolent but persistent clinical course.

Aziz et al.^27^ studied HPV genotype distribution in HIV‐positive and HIV‐negative women with cervical dysplasia. They found that 12.3% of HIV‐positive women presented with abnormal cytology results, either LSIL or ASC‐US. Of these abnormal cytology results, 50% were positive for HPV‐18 and 12.5% positive for HPV‐16. In another study by Cambrea et al.,^28^ HPV genotype distribution was studied in HIV‐positive women from southeastern Romania. HPV‐31 was found to be the most prevalent genotype. The only cytological abnormalities observed were ASC‐US and LSIL. HIV‐positive women with CD4+ T‐cell counts less than 200 cells/μL were nine times more likely to have HPV infection than those with CD4+ T‐cell counts greater than 200 cells/μL. The same study also found that the risk of HPV infection increased more than four‐fold in women with multiple sexual partners or early sexual debut. A key finding of our study is that the specific HPV genotype, rather than the degree of HIV‐related immunosuppression, was the primary driver of viral clearance timing in our cohort.

Specifically, patients infected with the HR‐HPV pool have a 58% lower probability than those infected with HPV 16/18 to clear their infection at a given time. The lack of correlation with CD4+ counts and HIV viremia suggests that in WLWH with stable immune function and high ART adherence, the natural history of HPV may be more influenced by virus‐specific biological factors than by residual HIV‐induced immune dysfunction.

Our study did not identify any statistically significant correlation between smoking status and clearance time of HPV. This result is consistent with recent literature. Indeed, in their recent systematic review on which investigated the relationship between smoking habits and HPV‐related cervical lesions, Zhao et al.^29^ found no significant relationship between current active smoking and HPV clearance. Active smoking is known to be a risk factor for HPV infection and its lesions. However, its relationship with HPV clearance is less clear. To date, no studies have investigated the potential impact of passive smoking on HPV‐related outcomes in WLWH.

Indeed, in HIV+ subjects, immune responses to HPV vaccines have been found to be suboptimal but improved after ART. Indeed, data suggest reduced antibody and memory B‐cell responses in HIV+ subjects compared to HIV‐negative subjects. Only a few studies have investigated HPV vaccine efficacy in PLWH. These studies showed reduced immune responses but suggested a better immune response in subjects with controlled HIV replication. Indeed, some authors suggested the potential efficacy of a fourth dose of vaccine in PLWH due to the moderate duration of antibody responses observed up to 5 years after vaccination.

To our knowledge, no previous studies have specifically investigated time to HPV clearance in WLWH. This study is one of the first attempts to investigate clinical and immunological factors associated with HPV clearance in a population with predominantly negative or low‐grade cytology and negative colposcopy.

The main limitations of our study include its retrospective design, the relatively small sample size (*n* = 51), the single‐center setting, and the potential for selection bias inherent to retrospective datasets, which may limit the statistical power and generalizability of our findings. Despite these limitations, the use of a Cox proportional hazards model to adjust for clinical and immunological confounders strengthens the reliability of our observation that viral genotype is a key determinant of clearance kinetics. Moreover, data on additional clinical factors potentially influencing HPV clearance, including immunosuppressive conditions, prior cervical treatments, and menopausal status, were not systematically available due to the retrospective design and were therefore not included in the analysis, representing additional potential sources of bias.

## 5 CONCLUSION

In this retrospective study, women living with HIV showed an HPV clearance time of approximately 2 years under antiretroviral therapy. Baseline positive high‐risk HPV genotypes correlated with increased time to HPV clearance, although no such association was seen with viremia and CD4+ T‐cell count. Considering the observed clearance pattern, it would be advisable to have an interval of 2 years between HPV tests in this population, which would improve patient compliance and optimize resource allocation in this at‐risk population.

## AUTHOR CONTRIBUTIONS

**Annunziata Carlea**: Investigation; data collection; formal analysis; writing – original draft. **Dario Colacurci**: Conceptualization; methodology; formal analysis; writing – original draft. **Giorgia D'Apice**: Investigation; data collection; formal analysis; writing – original draft. **Laura Letizia Mazzarelli**: Investigation; data collection; formal analysis. **Massimiliano Pellicano**: Investigation; data collection; formal analysis; **Luigi Falco**: Investigation; data collection; formal analysis **Ginevra Sepe**: Investigation; data collection; formal analysis. **Oriana Imperatore**: Investigation; data collection; formal analysis. **Giuseppe Maria Maruotti**: Conceptualization; writing – review and editing; supervision. **Maurizio Guida**: Conceptualization; methodology; writing – review and editing; supervision. **Laura Sarno**: Conceptualization; methodology; writing – review and editing; supervision.

## FUNDING INFORMATION

This research received no external funding.

## CONFLICT OF INTEREST STATEMENT

The authors declare no conflict of interest.

## ETHICS STATEMENT

This study was conducted in accordance with the ethical principles of the Declaration of Helsinki and was approved by the Campania 3 Ethics Committee (protocol no. 128/2026; approval date: 19 May 2026). Written informed consent was obtained from all participants prior to enrollment.

## Data Availability

The data that support the findings of this study are available on request from the corresponding author. The data are not publicly available due to privacy or ethical restrictions.
